# Recent Progresses in Optical Biosensors for Interleukin 6 Detection

**DOI:** 10.3390/bios13090898

**Published:** 2023-09-21

**Authors:** Marjan Majdinasab, Marc Lamy de la Chapelle, Jean Louis Marty

**Affiliations:** 1 Department of Food Science & Technology, School of Agriculture, Shiraz University, Shiraz 71441-65186, Iran; majdinasab@shirazu.ac.ir; 2 Institut des Molécules et Matériaux du Mans (IMMM—UMR 6283 CNRS), Le Mans Université, Avenue Olivier Messiaen, CEDEX 9, 72085 Le Mans, France; marc.lamydelachapelle@univ-lemans.fr; 3 BAE: Biocapteurs-Analyses-Environnement, University of Perpignan Via Domitia, 52 Avenue Paul Alduy, CEDEX 9, 66860 Perpignan, France

**Keywords:** interleukin 6, biosensor, optical biosensor

## Abstract

Interleukin 6 (IL-6) is pleiotropic cytokine with pathological pro-inflammatory effects in various acute, chronic and infectious diseases. It is involved in a variety of biological processes including immune regulation, hematopoiesis, tissue repair, inflammation, oncogenesis, metabolic control, and sleep. Due to its important role as a biomarker of many types of diseases, its detection in small amounts and with high selectivity is of particular importance in medical and biological fields. Laboratory methods including enzyme-linked immunoassays (ELISAs) and chemiluminescent immunoassays (CLIAs) are the most common conventional methods for IL-6 detection. However, these techniques suffer from the complexity of the method, the expensiveness, and the time-consuming process of obtaining the results. In recent years, too many attempts have been conducted to provide simple, rapid, economical, and user-friendly analytical approaches to monitor IL-6. In this regard, biosensors are considered desirable tools for IL-6 detection because of their special features such as high sensitivity, rapid detection time, ease of use, and ease of miniaturization. In this review, current progresses in different types of optical biosensors as the most favorable types of biosensors for the detection of IL-6 are discussed, evaluated, and compared.

## 1. Introduction

A biomarker is a measurable indicator of some biological condition and especially pathophysiological processes, which can be used to diagnose or prognosticate a patient, as well as to monitor disease progression or a patient’s response to treatment [1]. Among the different types of biomarkers (cellular, molecular, vesicular), proteins have been significantly investigated and their potential in diagnosing diseases has been determined. Cytokines are small proteins that play an important role in cell signaling, and are often used as biomarkers for disease monitoring such as liver diseases [2], cancer progression [3], and hepatic inflammations [4]. Particularly, interleukin-6 (IL-6) plays a significant role in the human immune system’s response to infection and cell damage, and is secreted into the serum by T cells and macrophages in acute and chronic inflammation [1].

**Figure 1:**
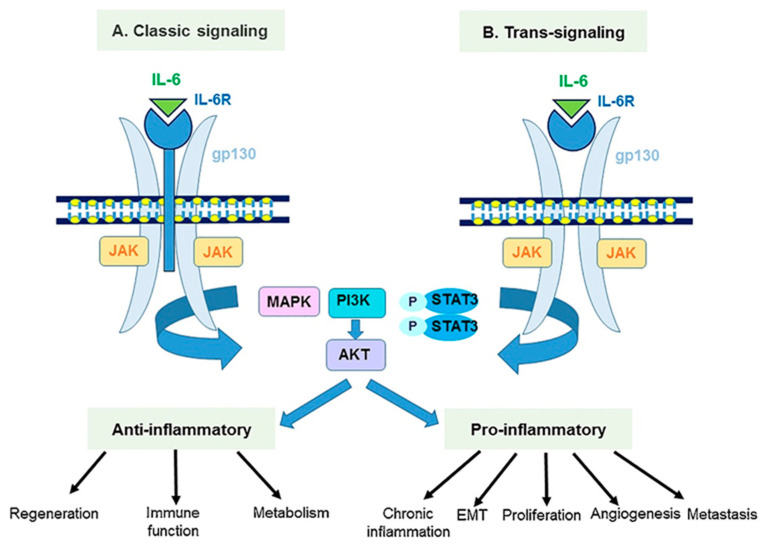
Interleukin-6 classic- and trans-signaling pathways. Reprinted from Ref. [13].

IL-6 is a pleiotropic cytokine polypeptide which can produce by various cells such as lymphocytes, endothelial cells, keratinocyte, neural cells, and bone cells, under stimulation by special inducers and at the site of inflammation [5,6]. Knowing the structure of IL-6 is important in analyzing its detection processes, as its properties such as isoelectric point (pI) and molecular mass affect its sensing function. IL-6 with a tertiary structure is composed of two glycoprotein chains and characterized by a four α-helical bundle structure [7]. The α-helix with terminal carboxyl group plays a key role in the receptor binding process [8]. Its molecular weight varies from 26 to 30 kDa, depending on cell-specific post-translational modifications [8]. Human IL-6 contains 212 amino acids [9]. The 29 amino acids serve as the N-terminal signal peptide, which is responsible for directing IL-6 to the correct orientation during receptor binding [10]. IL-6 communicates with cells by binding to its receptor, named the interleukin-6 receptor (IL-6R). IL-6R is an integral membrane protein containing a conserved region of 90 amino acids belonging to the immunoglobulin supergene family [8,11]. It is a three-domain specific receptor for IL-6 with a double helix structure and containing two anti-parallel fibronectin III type domains. IL-6R is only found on the surface of specific cells including macrophages, monocytes, hepatocytes, B cells, and T cells [10]. When IL-6 is inserted into the IL-6R binding pocket, another 130 kDa signal-transducing β-subunit glycoprotein 130, gp130, acts as a signal transducer, facilitating the formation of high-affinity extracellular receptor binding sites [8]. The gp130 is a general receptor for the IL-6 cytokine family and contains six β-sheet sandwich domains [11]. Therefore, in inflammatory disorders, IL-6 activates cells through a heterodimeric signaling complex including IL-6R and gp130, which is shared with several other cytokines [12]. The IL-6R is found in two forms, soluble (sIL-6R) and membrane-bound forms, which differentiates between IL-6 classic (through the membrane-anchored IL-6R) and IL-6 trans-signaling (through the sIL-6R). Formation of the IL-6 classic signaling, induced by IL-6 binding to IL-6R and then gp130, activates the JAK/STAT pathway in macrophages, monocytes, hepatocytes, B cells and T cells (classical signaling) to exert anti-inflammatory effects (Figure 1). On the other hand, IL-6 binding to sIL-6R and then gp130 activates the JAK/STAT pathway in non-IL-6R expressing cells (trans-signaling), resulting in pro-inflammatory responses. Finally, soluble gp 130 (sgp130) can bind to IL-6 and sIL-6R complexes to inhibit cellular IL-6 binding and regulate the trans-signaling pathway [10,13]. IL-6 receptors can be ideal biorecognition elements in different sensing techniques and as a suitable substitute for antibodies and aptamers in order to specific detection of IL-6. Although there are several recent studies on electrochemical biosensors based on IL-6 receptor as recognition element [14,15,16], what is missing from these specific elements is felt in studies related to optical biosensors.

There are several recent reviews that explain different aspects of IL-6 ranging from its biological function [8,10], detailed explanations of the activated signaling pathways [8,10,17,18], to therapeutic methods based on specific blocking of IL-6 [19,20,21].

Since IL-6 is involved in different biological processes including immune regulation, hematopoiesis, tissue repair, inflammation, oncogenesis, metabolic control, and sleep [9], it has a pivotal role in acute, chronic, and communicable diseases. Therefore, IL-6 can be considered a useful biomarker in diagnosis infection and cardiovascular diseases [22,23], different kinds of cancer [24,25], and other diseases such as lung fibrosis [26], chronic intestinal inflammation [27], acute kidney injury (AKI), chronic kidney disease (CKD) [28,29,30] and, recently, COVID-19 [31]. The reported value for IL-6 in the blood of healthy individuals is <10 pg/mL [32], and this level is increased in serum or blood of patients with different pathological problems [33]. The level of IL-6 in in a variety of pathological disorders is shown in Table 1.

**Scheme 1:**
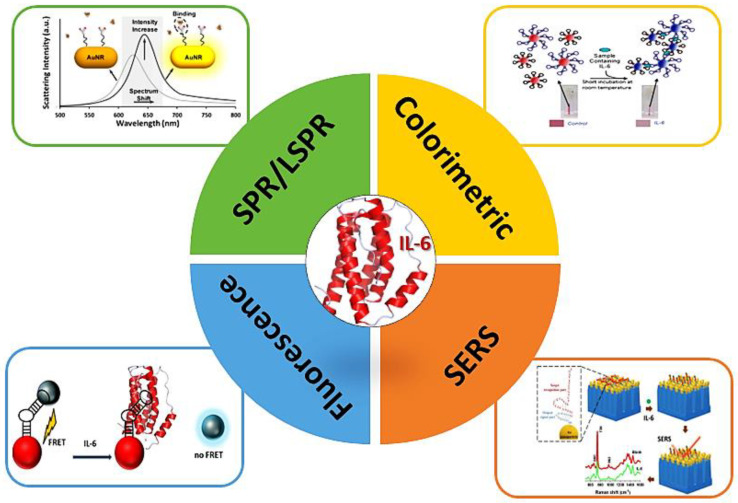
Different types of optical biosensors for IL-6 detection.

Laboratory techniques such as enzyme-linked immunoassays (ELISAs) and chemiluminescent immunoassays (CLIAs) are the most common conventional methods for IL-6 detection with high sensitivity (≈10–20 pg mL^−1^), specificity, and reliability [34]. Despite several benefits of these techniques, they are costly, time-consuming, require large sample volumes, and must be performed by skilled personnel [35]. On the other hand, Prompt diagnosis is essential for IL-6-related diseases such as sepsis, where diagnosis and administration of specific therapy within the first 6 h of disease onset can significantly improve patient outcomes. [36]. Consequently, the development of rapid, cost-effective, and user-friendly sensing techniques with high sensitivity and specificity, the possibility of miniaturization, and portability for the detection of IL-6 is and other important biomarkers is critical. Such sensing devices can also be useful for at-home monitoring of some diseases in which IL-6 is implicated. Based on the transduction method, biosensors can be categorized into five groups, including electrochemical, electrical, optical, piezoelectric, and thermal [37]. Optical biosensors have gained further advantage in biotechnology, disease diagnosis, medical devices, and environmental assessment, due to their selective, rapid, and highly sensitive measurements [38]. Optical biosensors are analytical devices that consist of a biorecognition sensing element integrated with an optical transducer system [39]. They emit an optical signal that is directly proportional to the analyte concentration. In a broad classification, optical biosensors are divided into two categories: label-free and label-based types. In label-free biosensors, the detected signal is directly produced by the interaction of the analyzed material with the transducer. In contrast, in the label-based biosensors, a fluorescent or a colorimetric label is used and the intensity of the fluorescence or colorimetric generated signal is proportional to the analyte concentration [39]. A variety of biomaterials, including antibodies, nucleic acids, enzymes, receptors, whole cells, and tissues can be used as biorecognition elements in optical biosensors [38,39]. Different major types of optical biosensors include surface plasmon resonance (SPR), fluorescence/luminescence, optic fiber, ring resonator, interferometer, optical waveguide, and photonic crystals. In this review, we focus on the main types of optical biosensors developed for IL-6 detection in recent years. Sensor design, performance of detection, and their advantages and disadvantages will be described. As far as we know, there has not been any comprehensive review in the field of different types of optical biosensors including SPR, colorimetric, fluorescence, and Raman-based biosensors for IL-6 detection (Scheme 1) in recent years. Therefore, this review can be a great collection of the main types of optical biosensors for readers. it is worth noting that, related to the IL-6 detection, optical biosensors are very important not only in laboratory research and development, but also in commercialized products, such that some of main detection strategies used in commercial biosensors for IL-6 are related to optical biosensors, among which we can mention colorimetric and fluorescence biosensors based on the lateral flow immunoassay (LFIA). Also, this article helps researchers in understanding the need to develop other types of optical biosensors, such as chemiluminescence and label-free Raman technology in the detection of IL-6, as well as the use of other types of nanomaterials as labels to increase the assay sensitivity and dynamic range of detection.

**Figure 2:**
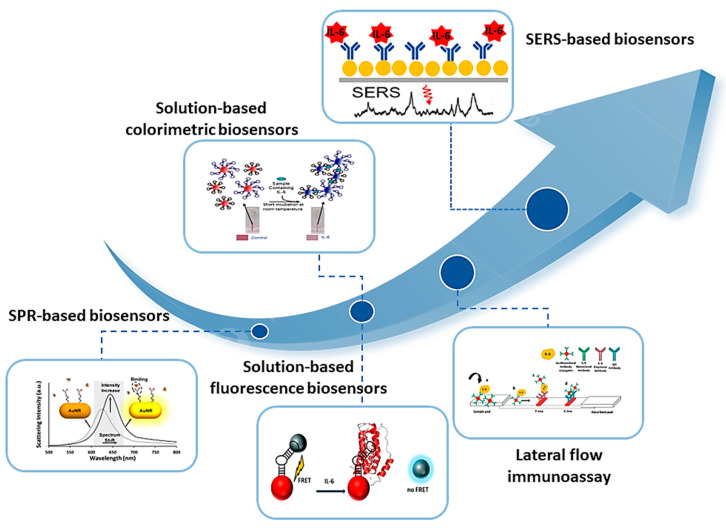
A roadmap of progress in optical biosensors for IL-6 detection.

A roadmap of progress in optical biosensors for IL-6 detection is shown in Figure 2. It can be observed that paper-based methods, such as lateral flow assays, as well as the SERS-based biosensors, are emerging and in progress methods for the detection of IL-6 in the future.

## 2. Surface Plasmon Resonance-Based Optical Biosensors

Among all types of optical biosensors, the most common, predominant, and extensively used technique is the surface plasmon resonance (SPR) optical biosensors, due to high sensitivity, robustness, cost-effectiveness, multiplexing, and diversity [40]. SPR is a phenomenon which occurs when the free electrons in the metal surface layer are excited by photons of incident light at a specific angle, causing an evanescent wave [41]. This causes the production of surface plasmons and thus reduces the intensity of the reflected light at a specific angle called the resonance angle [39]. This phenomenon is beneficial for monitoring changes in refractive index, because the evanescent wave is very sensitive to changes in the vicinity of the surface [41]. SPR occurs only at the nanometer scale in metals (gold and silver are preferred) and are classified into two categories: Localized Surface Plasmon Resonance (LSPR), when the phenomenon occurs on sub-wavelength-sized metal nanoparticles, and SPR, when it happens on thin metallic films [42]. The plasmon generated is directly dependent on the size of the nanoparticle or metal film and the material used. Considering the type of material, from a physical point of view, silver is seen to be better due to its more intense plasmon. However, gold is more used because of its chemically inert nature [41]. One of the methods benefiting from the characteristics of each material is the application of multilayer or core–shell nanoparticles [43]. In terms of SPR emission, a thickness of 40 nm has been found to generate the highest plasmon intensity, while for nanoparticles, the smaller the diameter, the lower the plasmon intensity [41].

Fluidic SPR is considered the most common SPR method in analytical applications [44]. The Kretschamann configuration of an SPR instrument is commonly applied. A biosensor is constructed on the prism, coated with nanometric-size metal gold or, more often, on a glass slide coated with the nanometric-size gold fixed on the prism by an immersion oil [45]. The fluid sample containing analytes flows through a measuring cell, pressurized by a buffer. The SPR signal is measured continuously. Converting the SPR signal into an image using a CCD camera is called SPR imaging (SPRi). In contrast to the fluid SPR assay, the SPRi array shows a high potential for the detection of molecular biomarkers [45]. The difference between the two methods is the presence of an aqueous solution during the measurement of the SPR method, while in the SPR array, the aqueous solution is removed before the measurement. The SPRi array technique uses the Kretschmann configuration, with the biosensor consisting of a gold chip fixed by oil immersion on a prism. The surface of the chip is designed as an array of measurement points separated by a polymer. The array is divided into nine measuring cells separated by a hydrophobic paint. Each cell includes dozens of measurement points. The chip architecture allows simultaneous measurement of nine separate samples. Using the SPRi array technique, a biosensor was developed for IL-6 determination in blood plasma [45]. The SPRi biosensor was designed onto a gold chip coated with a photopolymer and hydrophobic paint. Two different strategies were used for detection. In the first one, the mouse monoclonal anti-IL-6 antibody as the receptor was immobilized on the gold chip through the cysteamine linker. In the second strategy, galiellalactone (an IL-6 inhibitor) as the receptor was immobilized on the gold chip with octadecanethiol (ODM) as the linker. In the antibody-based biosensor, the linear response range was between 3 (limit of quantification: LOQ) and 20 pg mL^−1^, while in the case of the galiellalactone-based biosensor, a linear range between 1.1 (LOQ) and 20 pg mL^−1^ was obtained. Both biosensors were investigated to determine IL-6 concentration in blood plasma before and after resection of ovarian tumors and endometrial cysts.

**Figure 3:**
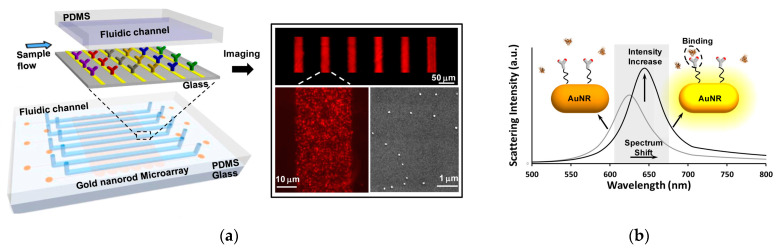
Schematic representation of the (**a**) LSPR microarray chip and its imaging under dark-field microscopy and scanning electron microscopy (SEM), and (**b**) and mechanism of detection relying on inducing a redshift and scattering intensity change in the longitudinal SPR. Reprinted from Ref. [47] with permission.

With recent advances in nanofabrication, plasmonic biosensing technologies have been developed into LSPR-based biosensors [46]. Using the LSPR technique, the surface binding of analyte molecules is recognized in real time from a shift in photon absorption and scattering characteristics of collectively oscillating conduction-band electrons, which are strongly localized on the surfaces of metallic nanoparticles [47]. In this context, a multiarray LSPR chip was developed for simultaneous detection of multiple cytokine biomarkers in low-quantity serum samples (1 µL) [47]. The microarray device was fabricated using easy-to-implement, one-step microfluidic patterning technique, and gold nanorods (AuNRs) were immobilized on the substrate surface within microfluidic channels using electrostatic interactions. Then, the nanorode microarrays were incorporated in a microfluidic chip with eight parallel detection channels comprising of input and output ports to load and wash reagent (Figure 3a). The patterned AuNR microarrays were conjugated with specific antibodies against cytokine molecules using EDC/NHS chemistry. Upon addition of analyte molecules and their binding to antibody-functionalized AuNRs, a redshift and changing in scattering intensity of longitudinal SPR was observed (Figure 3b). The limit of detection (LOD) for the six cytokines were determined in the range of 5–20 pg mL^−1^, and for IL-6 was 11.29 pg mL^−1^. The total time of all test steps was estimated to be about 40 min. An excellent correlation was obtained between the LSPR chip and ELISA method.

While antibodies are mainly used as detection elements, these very large (∼150 kDa) Y-shaped proteins show obvious disadvantages in probing nanosized cytokines (6–70 kDa) in a label-free LSPR assay design [48]. Therefore, development of new probes with smaller sizes and higher analyte-to-probe mass ratios to exceed the theoretical limit of LSPR bioassays. Nanobodies and peptides aptamers with high binding affinity to protein analytes have emerged as new biorecognition element. Peptide aptamers are short sequence of amino acids with a molecular weight of 3–5 kDa, which shows high affinity to the selected target. Using antibody-derived peptide aptamers (ADPAs) with very small dimensions (1.5 nm), He et al. (2022) developed a LSPR imaging (LSPRi) immunoassay for label-free detection of IL-6. For the biosensor fabrication, a polydimethylsiloxane (PDMS) layer with microfluidic channels was attached to the APTES-modified glass slides. AuNRs were loaded into the microfluidic channels and interacted electrostatically with APTES. The ADPAs was attached on the sensing surface through Au–S bonding. By using a dark field imaging system and a minimal sample of 3 μL, the immunosensor received label-free detection of IL-6 with a LOD down to 4.6 pg mL^−1^ and within a total assay time of 35 min. Compared to its antibody counterpart, the ADPA showed higher sensitivity whiling maintaining high specificity.

Representative examples of recently developed SPR-based optical biosensors for the detection of IL-6 have been listed in Table 2.

## 3. Colorimetric-Based Optical Biosensors

**Figure 4:**
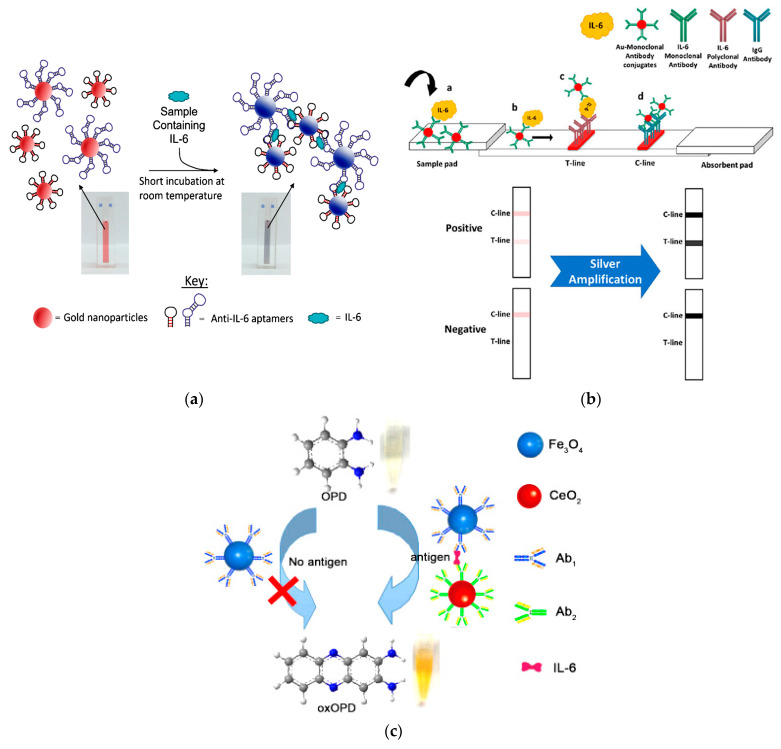
A schematic illustration of the (**a**) aptamer-AuNPs-based aggregation method for the detection of mouse IL-6; (**b**) LFIA incorporated with silver enhancement for the IL-6 detection in serum; and (**c**) sandwich immunoassay-based on magnetic nanoparticles and oxidase activity of ceria nanospheres. Reprinted from Refs. [33,54,68], respectively, with permission.

Colorimetric sensors/biosensors show promising potential for the detection of different analytes due to easy fabrication, rapid detection, low-cost, high sensitivity and selectivity, as well as easy measurement with the naked eye [52]. Colorimetric immunoassays are among the most widely used and sensitive methods in the detection of biomarkers. Especially after the emergence of nanomaterials, new opportunities have been provided in the further development of these methods. In this regard, gold nanoparticles (AuNPs) are the most commonly used nanomaterials in colorimetric sensors/biosensors due to their unique plasmonic characteristics, which enables them to aggregate and change color quickly under the influence of the changes in their surrounding environment and produce a colorimetric signal visible to the naked eye. Such biosensors are based on color changing of the AuNP colloidal solution due to the nanoparticle aggregation, and are defined as aggregation plasmonic sensors [53]. Giorgi-Coll et al. (2020) developed an optical aptasensor based on the aggregation of AuNPs conjugated with two complimentary “sandwich-type” aptamers, each with different IL-6 target regions [54]. Upon addition of IL-6 molecules, they bound to the aptamers on the AuNPs surface, resulting in aggregation of the AuNP, a color change from red to pink, and a corresponding change in the maximum absorption from 520 to 540 nm (Figure 4a). The test was completed in 5 min without any sample preparation steps. The LOD was obtained 1.95 µg mL^−1^ with a linear range from 3.3 to 125 µg mL^−1^. Development of portable and user-friendly instruments for highly sensitive and rapid detection of IL-6 is the focus of many researchers. Smartphone-based biosensors have attracted widespread attention and exhibited high potential as portable and cost-effective analytical devices for point-of-care (POC) diagnostics [55]. In this context, smartphones equipped with optical biosensors, especially colorimetric sensors, have attracted much attention [55]. In smartphone-based colorimetric biosensors, the color signal is converted into quantitative data using a smartphone as the most accessible reading tool. Therefore, smartphones can be suitable for mobile diagnostic and monitoring devices for POC testing, including paper-based sensors. Alba-Patiño et al. (2020) developed a paper-based colorimetric biosensor paired with smartphone for the rapid detection IL-6 as a major biomarker of sepsis in whole blood [56]. To fabricate the biosensor, captured antibodies were immobilized on the surface of filter paper by dropping. After adding IL-6 and binding to captured antibody, the detection antibody was included to form an immunocomplex. Finally, AuNP functionalized anti-rabbit IgGs were added to produce colored spots on the paper substrate. The colorimetric signals were evaluated with the mobile densitometry app. The biosensor was able to detect IL-6 with a LOD of 0.1 pg mL^−1^, a linear range of 0.001–10 pg mL^−1^, and a total assay time within 17 min. Moreover, it was able to detect an increase in IL-6 of only 12.5 pg mL^−1^ over baseline levels in whole blood with 99% confidence.

Lateral flow immunoassay (LFIA) is one of the most ideal and acceptable analytical techniques among paper-based point-of-care testing (POCT), because of several advantages such as low cost, user friendliness, and time saving. This technique exhibits high potential for detecting different analytes including disease biomarkers [57,58]. An LFIA strip commonly comprises four main parts: (1) the sample pad to which the sample (liquid) is inserted, (2) the conjugation pad consisting of capture bioreceptor labelled with mostly nanomaterials, (3) the nitrocellulose membrane on which the test (T) and control (C) lines are sprayed, and (4) the absorbent pad that plays the role of a wick and collects excess liquid [57]. LFIAs are usually designed based on two main detection strategies, including sandwich-type and competitive-type assays. A sandwich-type LFIA is the most-preferred format for the detection of larger-sized analytes (>1 kDa), such as proteins, antibodies, bacteria, and cells. In this format, the analyte is sandwiched between the capture antibody and the labelled antibody on the test line generating a visual color signal directly proportional to the analyte concentration. The Competitive format is generally performed to detect low molecular weight analytes which have an antibody binding site. In this format, the target molecule in the sample and the target/target analog immobilized on the test line compete for binding to labelled antibodies, resulting in the appearance of a visual color signal inversely proportional to the target concentration [59].

Since the IL-6 seems to be related to respiratory failure, it can be considered as a biomarker of COVID-19 detection [60]. For early recognition of COVID-19-infected patients under imminent risk of acute respiratory failure, a sandwich format of IL-6 LFIA strip accompanied by a spectrum-based optical reader was developed [61]. The test strip was designed based on immobilization of 15 nm AuNPs conjugated with anti-IL-6 antibody onto conjugated pad. The nitrocellulose membrane was coated with anti-mouse IgG monoclonal antibody and the quality control antibody at the T and C lines, respectively. Under applying the sample on the strip, the human IL-6 antigen in the sample formed an antigen complex with the anti-IL-6 antibody–AuNP conjugates. Then, these complexes were captured in the T line and displayed colored bands. The spectrum-based optical reader provided continuous spectral reflectance values with high resolution when reading the result of the T line through an optical module. Clinical samples related to the COVID-19 patients were analyzed by developed test strips coupled with an optical reader, and satisfactory results were obtained, which provided a promising POC testing method for early detection of COVID-19 patients at risk of acute respiratory failure.

AuNPs, as the most common labels in LFIA, are not fully able to cover the desired need for sensitive detection specially in relation to highly sensitive quantitative analysis of low-abundance biomarkers such as cytokines [33]. In order to overcome this weakness, several signal amplification techniques have been developed, enabling sensitive colorimetric detection of different analytes through AuNP-based LFIAs. Silver enhancement is one of those methods that is based on the nucleation of silver on the gold surface in the test or control lines of LFIA strips and increases the intensity of the obtained colorimetric signal. Since the target detection limit for IL-6 is very low (i.e., in the pg ml^−1^ range), Rahbar et al. (2021) employed a silver enhancement technique incorporated with AuNPs-based LFIA to reach the desired sensitivity [33]. In this study, AuNPs were used as label in conjugation with the specific IL-6 antibody (Figure 4b). The T line was comprised of the biotinylated polyclonal human IL-6 antibody and streptavidin, while goat anti-mouse IgG antibody was directly dispensed at the C line. After conducting the general sandwich test, a silver enhancement solution containing a 1:1 ratio of silver nitrate and hydroquinone was applied to the strips to enhance the produced colorimetric signals. With this procedure, IL-6 was detected in both buffer medium and serum samples with a LOD as low as 1 and 5 pg/mL, respectively.

In addition to signal amplification techniques, the application of other nanomaterials such as Pt nanoparticles with excellent optical properties [62] or nanoscale materials with natural enzyme-like activities (Nanozymes) and catalytic properties have attracted interest in colorimetric assays. Unlike natural enzymes, nanozymes provide uninterrupted biocatalytic activity even under extreme conditions of pH, temperature, and resistance to protease digestion [63]. Nanomaterials with peroxidase activity have received a lot of attention in biomedical applications for the past few years. Various nanomaterials such as metal/metal oxide nanoparticles [64,65], carbon nanomaterials [66], and different metal–organic frameworks (MOFs) [67], etc., represent excellent catalytic activities by mimicking the functions or structures of natural peroxidase enzymes, which are widely used to oxidize organic substrates. In this regard, a magnetic colorimetric immunoassay-based on the oxidase activity of ceria nanospheres was developed for IL-6 detection [68]. The CeO_2_ nanospheres were employed to label the signal antibodies (Ab_2_), while Fe_3_O_4_ nanospheres were used to immobilize capture antibodies (Ab_1_) (Figure 4c). In the presence of IL-6, an immunocomplex was formed between Fe_3_O_4_-Ab_1_ and IL-6. After magnetic separation, CeO_2_-Ab_2_ was included to form a sandwich complex of Fe_3_O_4_-Ab_1_/IL-6/CeO_2_-Ab_2_. Following magnetic separation and o-phenylenediamine (OPD) addition, ceria spheres with excellent oxidase activity directly catalyzed the oxidation of substrate OPD to a stable yellow product, 2,3-diaminophenazine (oxOPD). The absorbance of oxOPD at 448 nm was related to the IL-6 concentration. The immunoassay showed a low LOD of 0.04 pg mL^−1^ and a linear range of 0.0001–10 pg mL^−1^. 

Representative examples of recently developed colorimetric-based optical biosensors for the detection of IL-6 have been listed in Table 3.

## 4. Fluorescence-Based Optical Biosensors

Fluorescence biosensors with high sensitivity are promising sensing platforms in clinical monitoring. Fluorescence is a two-step chemical phenomenon that involves the absorption of shorter wavelength light by a fluorophore (excitation), followed by the release of some of the absorbed energy as longer wavelength light (emission) [72]. Fluorescence-based biosensors are advantageous because of several key aspects such as sensitivity, signal detection limits, and accuracy [73]. Organic dyes are generally employed as tags in fluorescence-based detection methods due to easy availability, low cost, and more versatility. Most generally used fluorescent dyes are based on cyanine structure or xanthene dyes. Fluorescein and rhodamine are the first organic dyes used for fluorescent labelling. Despite the many advantages of these dyes, they suffer from some weaknesses such as light bleaching, pH sensitivity, and hydrophobicity [74]. With the emergence of nanotechnology and the production of nanomaterials with fluorescence properties such as quantum dots (QDs), carbon nanotubes (CNTs), carbon dots (CDs) and up-conversion nanoparticles, many challenges of these organic materials were overcome [73,74]. One of the interesting materials used as a fluorescent tag is Europium(III) particles with unique luminescence characteristics, such as long fluorescence lifetimes, narrow emission spectra, large shifts, and a very sharp emission profile with a full width at half maximum of about 10 nm, and emission from atomic states [75]. All these features contribute to high sensitivity and accuracy in an assay. Using these excellent properties, Eu nanoparticles (EuNPs) were used as fluorescent label in combination with a time-resolved LFIA for IL-6 detection [32]. For developing this double-antibody sandwich assay, IL-6 monoclonal antibody (mAb) and chicken IgY were conjugated with EuNPs, and a mixture of them (IL-6-mAb-EuNPs and chicken IgY-EuNPs) was dispensed onto conjugate pad. A second IL-6 mAb-2 and anti-chicken IgY were sprayed onto the nitrocellulose membrane as the T line and the C line, respectively. Upon addition of sample containing IL-6 and its migration toward conjugate pad, the immunocomplex of IL-6-mAb-EuNPs/IL-6 was formed. After reaching this complex to the T line, it was captured by the IL-6 mAb-2 and formed IL-6-mAb-EuNPs/IL-6/IL-6 mAb-2. Chicken IgY-EuNP complexes continued to move along the membrane and were captured in the C line by the anti-chicken IgY to form Chicken IgY-EuNPs/anti-chicken IgY complex, which served as the internal control. After the completion of the reaction, the test strip was analyzed with a portable fluorescence reader by measuring the fluorescence intensity of the T line (I_T_) and the C line (I_C_). The concentration of IL-6 in the samples was directly proportional to the ratio of I_T_/I_C_. This test strip showed a LOD of 0.37 pg mL^−1^ with a wide linear range of 2–500 pg mL^−1^. 

**Figure 5:**
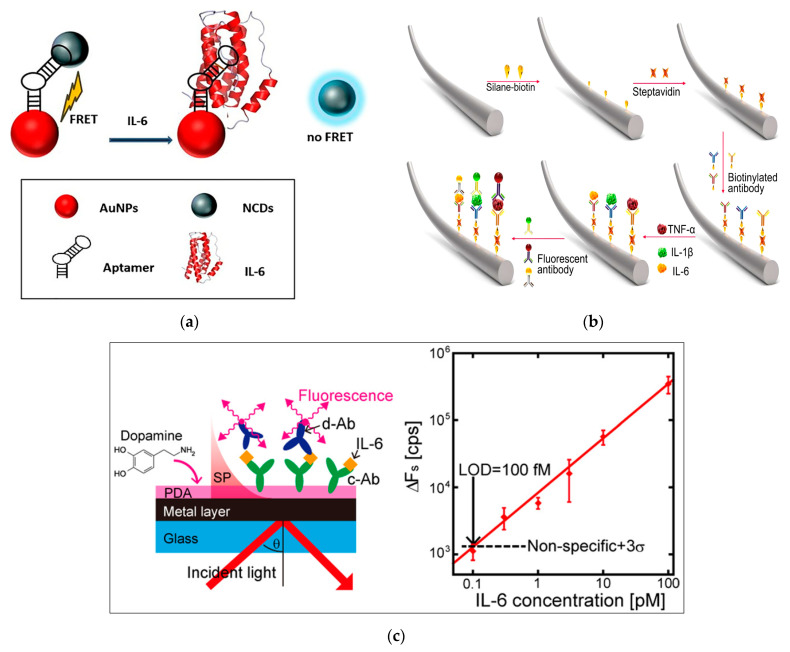
A schematic representation of (**a**) A FRET-based aptasensor for IL-6 detection; (**b**) a fiber-based multiplex fluorescence immunosensor; and (**c**) a sandwich immunosensor-based on polydopamine thin film as protein linker in combination with SPFS technique. Reprinted from Refs. [79,83,84], respectively, with permission.

Carbon quantum dots (CQDs) are considered as a new class of fluorescence nanomaterials. They show a lot of unique advantages compared to traditional fluorescent nanomaterials, such as excellent photostability, good solubility, low toxicity, and considerable biocompatibility, as well as their small size [76]. Due to these advantages, CQDs are considered interesting fluorescent labels and ideal alternatives to conventional fluorescent compounds, which can be used in a variety of detection purposes such as biomarker monitoring. The two main drawbacks of CQDs including low performance and trace of functional groups on their surface restrict the fluorescence efficiency and application of CQDs [77]. Doping of CQDs by nitrogen, sulfur, and other elements, can improve their spectral characteristics such that N-doped CQDs (N-CQDs) and S-doped CQDs (S-CQDs) exhibit higher fluorescence intensity [78]. Using N-CQDs, Mahani et al. (2022) developed an aptasensor based on the Forster resonance energy transfer (FRET) phenomenon for IL-6 detection as a biomarker of COVID-19 (Figure 5a) [79]. FRET is the transfer of energy from a fluorescent donor in an excited state to an acceptor in the ground state. This phenomenon is widely used in fluorescence sensing assays due to its advantages in investigating molecular interactions in real time, being relatively simple and easy to use, and having high sensitivity and spatial resolution [79]. For the fabrication of aptasensor, a DNA aptamer was modified with N-QCDs at one end and AuNPs at another end to prepare a donor–quencher pair. In the absence of IL-6, the AuNPs acting as acceptors were effectively located close to the donors (N-CQDs), which led to a reduction in the emission intensity of the N-CQDs resulted from their highly overlapping spectra. The interaction of IL-6 with the detection probe caused aptamers to preferentially bind to IL-6, resulting in structural and conformational changes that dissociates N-CQDs from aptamers, such that fluorescence emission was recovered. The designed aptasensor with high sensitivity and specificity showed a LOD of 0.82 pg mL^−1^ and a linear range from 1.5 to 5.9 pg mL^−1^.

Different strategies can be used to increase the fluorescence intensity in response to minimum detectable concentration, such as conjugate decoration with several fluorescent molecules [80] and the use of a composite tapered optic fiber [81].

Fiber optic cables, as one of the components of biosensors, are used as extenders of sensor amplifier systems to increase their sensitivity to signals, especially in inaccessible areas, and increase the range of the sensor. Fiber optic biosensors can be used both invasively and non-invasively in medical applications. They have been employed in different applications such as gas, tissue, or body fluid analysis, as skin electrodes, as catheters, and as endoscopic tools [82]. Fiber optic biosensors are distinguished from other biosensors by the optical-based transducer that utilizes absorption, reflectance, luminescence, refractive index, and scattering of light to transform the signal for processing (Figure 5b). In this regard, a fluorescent immunosensor on optical fiber was designed for the multiplex detection of cytokines, including interleukin-1β (IL-1β), IL-6, and tumor necrosis factor-α (TNF-α). To develop the biosensor, the streptavidin-modified fiber surface was coated with biotinylated captured antibodies against three different cytokines. On the other hand, three different fluorescent magnetic beads, including fluorescent green magnetic beads, fluorescent orange magnetic beads, and fluorescent red magnetic beads, were used for the conjugation with detection antibodies. After complex formation between immobilized antibodies, cytokine molecules, and conjugated detection antibodies, the final fiber biosensor was imaged using a laser scanning confocal microscope. This multiplex immunosensor was applied for the detection of three cytokines with the LOD of 12.5 pg mL^−1^ and a linear range of 12.5–200 pg mL^−1^ [83]. 

A dual amplification strategy, including the application of polydopamine thin film as a protein linker in combination with surface plasmon-enhanced fluorescence spectroscopy (SPFS), was used for sensitive detection of IL-6 [84]. In this platform, PDA thin film was deposited on glass substrate coated with gold (Figure 5c). The captured antibody was directly attached to the sensor surface without using any coupling agent. Then, IL-6 was detected by SPFS using a sandwich assay format upon addition of secondary detection antibody labelled with Alex Fluor 647. The use of PDA for functionalization of the sensor chip increased the sensitivity and inhibited nonspecific adsorption of the detection antibody onto the sensor surface. A LOD of 2 pg mL^−1^ with a linear range of 2–2372 pg mL^−1^ was obtained.

Representative examples of recently developed fluorescence-based optical biosensors for the detection of IL-6 have been listed in Table 4.

## 5. Surface-Enhanced Raman-Scattering-Based Optical Biosensors

Raman Spectroscopy is a non-destructive analytical technique which provides a unique vibrational fingerprint spectrum of a molecule [88]. The principle of detection is the interaction of light (from a high intensity laser light source) with the chemical bonds within a material. Since the Raman phenomenon results from inelastic scattering of light, the spectral signal intensity is very weak, and this major drawback prevents the technique from being used for detection applications [88]. This limitation was overcome by the discovery of the Surface-Enhanced Raman Scattering (SERS) phenomenon, where a low Raman cross-section can be enhanced when the molecules to detect are adsorbed on rough metal surfaces or by nanostructures [89]. Therefore, SERS is known as a powerful technique with high efficiency to enhance the Raman signal of chemical and biological species, and is capable of observing and identifying very low concentration of molecules, which paves the way for single-molecule sensing [90,91]. Due to the high sensitivity of SERS-based assays, this technique can be extensively used for detection of very low quantities of desired analytes in medical diagnoses [92,93], the food industry [94,95], and environmental monitoring [96,97]. SERS-based detections can be performed in two formats, direct and indirect. Direct detection is based on ability of SERS technique for providing unique molecular fingerprints of molecules adsorbed on a nanostructured metallic surface, as well as other types of nanomaterials [98]. However, direct detection of some molecules such as proteins by the SERS technique is very difficult, due to their low Raman scattering cross-section and the lack of the presence of the chromophore moiety in the molecular structure to generate a strong SERS signal [34]. Therefore, the combination of highly specific biomolecules (e.g., antibodies and aptamers) with SERS for indirect sensing can greatly improve the use of the SERS technique in highly selective quantification of such analytes [99]. In the indirect SERS assay, two strategies can be used: label-based and label-free. The label-based strategy is considered an indirect method that relies on the use of SERS tags. The use of SERS labels results in highly sensitive detection of biological analytes by reporting the Raman spectrum of the reporter molecule in close proximity to the SERS substrate [100]. Compared to the label-based strategy, the label-free approach does not require a Raman reporter molecule and can directly report the intrinsic SERS fingerprint relies on the mutual interaction of target molecule with the SERS substrate [101]. 

**Figure 6:**
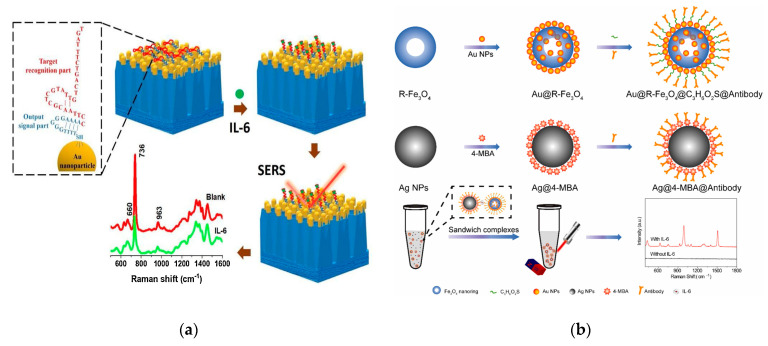

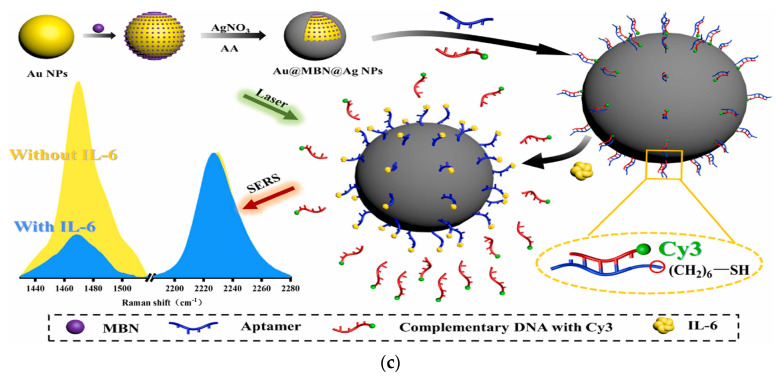
A schematic representation of (**a**) AuNP array functionalized with aptamer for label-free detection of IL-6; (**b**) a sandwich-based immunoassay using Fe_3_O_4_ nanoring for IL-6 detection; and (**c**) a SERS-based self-calibrating aptasensor using a pair of Raman tags. Reprinted from Refs. [34,102,103], respectively, with permission.

Combining SERS with aptamer as recognition element can be a very promising method for label-free, sensitive, and specific detection of various target molecules. The principle of label-free detection using the aptamer itself as an intrinsic Raman reporter is as follows: upon interacting with the target molecule, the aptamer folds into a different three-dimensional configuration, such as a hairpin or G-quadruplex, and this conformational changes close the surface of the SERS substrate results in a corresponding change in output Raman signals, with which the analyte can be specifically measured with high sensitivity [102]. With this concept, a label-free SERS assay was developed for IL-6 detection using an IL-6 specific aptamer attached to the AuNPs array as a sensitive probe [102]. For the design of this aptamer–SERS biosensor, the original sequence of aptamer was modified with extra thymine, guanine, and adenine bases near thiol linker (Figure 6a). The T fragment functioned as a spacer between the surface of AuNPs and the aptamer to provide specific flexibility and inhibit non-specific adsorption of the aptamer, while the SERS intensity ratio of G and A operated as an output signal in the aptamer-IL-6 interaction. Upon addition of aptamer into serum containing IL-6, the 5′ loop of the aptamer was opened and the aptamer could adopt a vertical stretched position, causing a decrease in the adenine signal at 736 cm^−1^. Therefore, IL-6 could be detected by observing the change in the I_660_/I_736_ ratio of the SERS intensity derived from the guanine and adenine bases in the aptamer sequence. This label-free assay exhibited a LOD of 0.8 pM and a linear range of 10^−12^–10^−7^ pM. It was applied to detect IL-6 in mice serum with high selectivity.

Since the application of a SERS tag results in a highly sensitive detection, indirect label-based SERS can be useful for analytes where detection of small amounts is of particular importance. Among these analytes is IL-6, which usually requires a detection limit of ppb. For this reason, most of SERS-based IL-6 detection are performed with the aid of SERS tags. For example, Xie et al. (2023) developed a SERS-based sandwich immunoassay for the detection of IL-6 in serum using Fe_3_O_4_ nanoring, silver nanoparticles (AgNPs), AuNPs, and 4-mercaptobenzoic acid (4-MBA) as the SERS signal molecule (or SERS tag) [103]. As shown in Figure 6b, a Fe_3_O_4_ nanoring with a high specific surface area and strong magnetic property was functionalized with AuNPs and specific antibodies, and used as capture substrate. AgNPs modified with 4-MBA and antibodies were used as probe. Upon addition of the target molecule to the solution, it bound to antibodies in both probe and capture substrates to form a sandwich-like structure. SERS detection was performed after enrichment by ferromagnetism, and detection of IL-6 based on the signal of the probe molecules was successfully carried out. A LOD of 0.028 pg mL^−1^ and the linear range of 0.1–1000 pg mL^−1^ was obtained. The proposed immunoassay exhibited very high sensitivity and selectivity, and only required a very small amount of sample. 

In another study, a self-calibrating aptamer biosensor using a recognition-release mechanism was developed for IL-6 detection [34]. For the fabrication of aptasensor, AuNPs functionalized with mercaptobenzonitrile (MBN) Raman tag were coated with AgNPs to obtain a Au@MBN@AgNPs core–shell structure (Figure 6c). Then, the core–shell structure was modified with IL-6 aptamer (ssDNA1) and complementary pairing with the complementary strand (ssDNA2) labeled with cyanine 3 (Cy3). In the presence of IL-6, ssDNA1 could specifically bind to IL-6 and release ssDNA2-Cy3 conjugate to attenuate Cy3 signaling. The internal standard molecule MBN with a signal in the biological Raman quenched region (1800–2800 cm^−1^) was embedded into the core–shell structure in order to minimize the error caused by the experiment, which could prevent the effect of Raman signals of different biomolecules in the complex environment. Therefore, the stability of the assay results was improved using the internal standard molecule as the calibration peak. Using this strategy, a minimum LOD of 0.056 pg mL^−1^ and a linear range of 10^−9^ to 10^−5^ mg mL^−1^ was obtained.

Dual-mode sensors are very interesting for the joint detection of disease biomarkers by SERS or other types of sensing techniques. Generally, in SERS-based assays for multiplex detection, application of more than one Raman reporter molecules is necessary. In this regard, an important point to consider is selection of SERS probes with characteristic peaks in the Raman-silent region that do not interfere with each other. Based on this concept, a SERS sandwich-like method combining multisite boronic acid-functionalized magnetic nanomaterials (MBMNPs) and interference-free probes, including 4-mercaptobenzonitrile (4-MP) and ethynylbenzene (EB) reporters conjugated with antibodies as bio-capture and bio-recognition elements, was developed for joint detection of the sepsis biomarkers IL-6 and procalcitonin (PCT) [104]. For both core–shell interference-free SERS probes, AuNPs and highly SERS-active silver were selected as the substrates. The IL-6 antibody was immobilized on the surface of Au@4-MP@Ag, while PCT antibody was bound by dopamine quinone on the Au@Ag@polydopamine (PDA)-EB surface. The 4-MP and EB showed characteristic peaks at 2224 cm^−1^ and 1987 cm^−1^,respectively, in the Raman-silent region. Upon addition of target analytes, a sandwich complex was formed between MBMNPs, target molecules, and Raman probes Au@4-MP@Ag and Au@Ag@PDA-EB. The ultrasensitive and accurate multi-target joint detection of sepsis was carried out by a portable Raman spectrometer. The SERS immnosensor exhibited the LODs of 0.584 and 2.99 pg mL^−1^ for IL-6 and PCT, respectively. The response curves were obtained in the range of 1–5000 pg mL^−1^ for IL-6 and 10 pg mL^−1–^50 ng mL^−1^ for PCT.

Representative examples of recently developed SERS-based optical biosensors for the detection of IL-6 have been listed in Table 5.

## 6. Conclusions and Future Perspectives

IL-6 is an important biomarker involved in many diseases. Its pathological level varies from 2.45 and 500,000 pg mL^−1^ depending on the biological fluid, type, and severity of the disease. During recent years, various types of optical and electrochemical biosensors have been developed to detect this crucial biomarker (for more information, refer to references [10]). The main goal of all these studies has been to develop biosensors with high sensitivity and accuracy, high selectivity, integrability, and ease of use, and applicability in complex biological fluids. An important factor to consider here is that the detection limit of the developed biosensor should be within the range of prognostic levels of IL-6. Moreover, the sensor should be able to be used in complex biological fluids easily, without the need for complex sample pre-treatment and without interfering with interfering agents. Currently, apart from ELISA-based diagnostic kits, the only other commercialized method for IL-6 detection is the immunofluorescence assay (e.g., AccuDx CQ IL-6 from Accurex Co.(Mumbai, India) or RapidFor from Vitrosens Co. (Istanbul, Turkey)), which is a successful example of optical biosensors based on LFIA. This fast test kit is able to detect IL-6 in human serum, plasma, whole blood and peripheral blood samples within 15 min. The AccuDx CQ IL-6 sandwich LFIA test uses an anti-human IL-6 monoclonal antibody I conjugated with fluorescence latex coated on the junction of nitrocellulose membrane and sample pad, and another anti-human IL-6 monoclonal antibody II coated on the test line. The fluorescence intensity of test line increases in proportion to the amount of IL-6 in sample. After sample addition and immunoreaction, the test strip is inserted into AccuDx CQ Immunofluorescence Quantitative Analyzer to read results. The concentration of IL-6 in sample is measured and displayed on the screen. The value is stored in AccuDx CQ and available for downloading. The results can be easily transmitted to the laboratory or hospital information system. However, the assay suffer from a narrow dynamic range. As shown in Table 1, Table 2, Table 3 and Table 4, by comparing all the types of optical biosensors developed for the detection of IL-6, it can be concluded that almost all of them have good sensitivity, but in the meantime, SERS-based biosensors, in addition to having the lowest detection limit, also show a wider dynamic range. Although Raman is a new technique that has received a lot of attention from researchers in recent years, and it still faces challenges such as low reproducibility and difficulty in applying it to biological samples, it can be hoped that by removing these obstacles, this method become one of the best detection techniques with high sensitivity and wide dynamic range. In this regard, miniaturization and development of portable Raman systems in order to facilitate the diagnosis process in the clinic, emergency room, or even at home is of particular importance. Among different strategies of SERS-based biosensors, label-based methods (using Raman tag) have been widely used and only a handful of label-free and fingerprint-based methods have been investigated, while label-free methods can be less expensive, simpler, and faster compared to using Raman labels. Concerning other types of optical biosensors, fluorescent biosensors with high sensitivity are promising in response to minimum detectable concentration. These biosensors show high sensitivity and stability by using the unique features of fluorescent nanomaterials and have a high potential for commercialization.

In general, by reviewing the literature, it can be observed that a few studies have been performed in the field of IL-6 detection, and there is still a lot of work to be performed in the field in terms of both the variety of detection methods and the improvement of sensitivity and dynamic range. For example, in recent years, there is no research on the detection of IL-6 using chemiluminescence biosensors. On the other hand, it seems that the methods based on lateral flow immunoassay and SERS are more welcomed by researchers. In relation to the recognition element, antibodies have been the most used elements in biosensors for IL-6 detection, while in addition to antibody and aptamer, other recognition elements such as molecularly imprinted polymer (MIP) with lower cost, reusability, and easy functionalization can be integrated into sensor platform instead of widely used antibodies or even aptamers.

Given the common role of IL-6 in many pathologies, diagnosis based on IL-6 levels alone is generally impractical. The design and development of multiplexed sensors for the diagnosis of specific diseases facilitates such diagnosis. In this regard, multiplexed sensors can combine with machine learning methods in order to facilitate fast and accurate interpretation of sensor outputs. Machine learning is a subfield of artificial intelligence that offers another method to obtain insight into complex data. Machine learning-assisted biosensors can be applied in complex environments and without having the features of a laboratory study [108]. Machine learning techniques such as artificial neural networks (ANN) are emerging as promising approaches for data analysis, and can be integrated with sensors to improve disease diagnosis and prognosis. Due to the variable level of IL-6, as a common biomarker in many diseases, in different people and biological fluids, the application of machine learning methods to analyze the signals of IL-6 sensors improves the interpretation of such complicated results. ANNs are a subfield of deep learning techniques that employ mathematical operations and self-learning methods to extract beneficial information from data sets. Moreover, by integrating machine learning analysis tools and multiphotonic effects to increase applications in optical biosensors, the potential exists for better interpretation of biological factors. In conclusion, the integration of optical biosensors for IL-6 with advanced machine learning algorithms provides significant advantages in terms of rapid, accurate, and reliable disease monitoring and diagnosis.

## Figures and Tables

**Table 1:** IL-6 level in different pathological disorders. Reprinted from Ref. [8].

Pathological Disorder	IL-6 Level (pg mL^−1^)
Alzheimer’s Disease	85–567
Meningitis	450–32,000
Myocardial Infarction	28.5–46.5
Cardiac Myxoma	>56
Multiple Myeloma	5–33
Burkitt Lymphoma	100.3
Post-Transplant Lymphoproliferative Disease/PTLD	143–11,020
Cachexia	100
Rheumatoid Arthritis	17
Psoriasis	30,000
Hepatitis B Virus Infection/Hepatocyte Carcinoma	7–18.9
Sepsis	5000–100,000

**Table 2:** Representative examples of recently developed SPR-based optical biosensors for the detection of IL-6.

Type of Biosensor	Strategy of Detection	LOD	Linear Range	Matrix	Advantages	Disadvantages	Ref.
SPR	SPRi array technique and immobilization of antibody or IL-6 inhibitor on the surface of gold chip	3 pg mL^−1^ (antibody), 1.1 pg mL^−1^ (inhibitor)	3–20 pg mL^−1^ 1.1–20 pg mL^−1^	Plasma	High sensitivity and selectivity, good precision and recovery, rapid incubation time (10 min), label-free assay, real time monitoring	Narrow linear range, the need for spectrophotometer device	[45]
LSPR	Microfluidic channel arrays with immobilized gold nanorods conjugated with antibody	11.29 pg mL^−1^	nr *	Serum	Label-free assay, high sensitivity and selectivity, multiplex assay, no cross-reactivity for other cytokines, high accuracy, the need for a small sample volume, label-free assay, real time monitoring	Non determination of linear range, long incubation time (30 min), the need for spectrophotometer device	[47]
LSPR	Microfluidic channel arrays with immobilized gold nanorods conjugated with antibody-derived peptide aptamers	4.6 pg mL^−1^	5 to 1 × 10^6^ pg mL^−1^	Serum	High sensitivity and selectivity, label-free assay, the need for a small sample volume, total assay time of 35 min, high consistency with the “gold standard” ELISA, label-free assay, real time monitoring	The need for spectrophotometer device, low reproducibility in complex biological medium	[48]
LSPR	A single-cell secretion detection chip consisting of microwells, each well is surrounded by Au nanopillars capable of LSPR measurement, modification of Au nanopillar with IL-6 antibodies for the direct detection of single-cell secreted IL-6 via LSPR absorbance peak shift	Single-cell cytokine secretion	-	-	High specificity, cost-effective fabrication, label-free assay, real time monitoring	Non-determination of LOD and linear range, the need for spectrophotometer device, non-application	[49]
SPR	Direct immobilization of primary antibody through mixed self-assembled monolayer (SAM) using mercaptoundecanoic acid (MUA) and 6-mercapto-1-hexanol (MCH) on a gold surface, and indirect antibody immobilization to the Fc-binding domain of protein G on the mixed SAM; a sandwich complex formation between primary antibody, IL-6 and secondary antibody	1.3 ng mL^−1^—Direct 5.7 ng mL^−1^—Indirect	0.78–12.5 ng mL^−1^	-	High specificity, label-free assay, real time monitoring	Low sensitivity, narrow linear range, not applicable in the real sample, the need for spectrophotometer device, non-application	[50]
LSPR	Antibody immobilization on the surface of gold-capped nanopillar-structured cyclo-olefin-polymer (COP) film and observation of peak red-shift in the transmittance spectrum after IL-6 binding	10 ng mL^−1^	-	-	Label-free assay, real time monitoring, high specificity	Low sensitivity, non, determination of linear range, not applicable in the real sample, the need for spectrophotometer device, non-application	[51]

* nr: not reported.

**Table 3:** Representative examples of recently developed colorimetric-based optical biosensors for the detection of IL-6.

Type of Biosensor	Strategy of Detection	LOD	Linear Range	Matrix	Advantages	Disadvantages	Ref.
Colorimetric/solution-based	Aggregation of AuNPs conjugated with two complimentary “sandwich-type” aptamers and color change from red to pink	1.59 µg mL^−1^	3.3–125 µg mL^−1^	A mixed protein solution	High specificity, short incubation time (5 min)	Low sensitivity, narrow linear range, non-application in the real sample, no very obvious color change (red to pink)	[54]
Colorimetric/paper-based	Immunocomplex formation between capture antibody on the surface of paper, IL-6, detection antibody, and AuNPs functionalized anti-rabbit IgG	0.1 pg mL^−1^	0.001–10 pg mL^−1^	Whole blood	High sensitivity, wide linear range, application in complex matrix of blood, short assay time (17 min), smartphone-based signal quantification	The need for several washing steps like ELISA, which makes it difficult to use	[56]
Colorimetric/LFIA	Immobilization of anti-IL-6 antibody-AuNPs conjugates onto conjugated pad, anti-mouse IgG monoclonal antibody and the quality control antibody at the T and C lines, respectively	cutoff level of 76.85 pg mL^−1^	nr *	Serum	High specificity, easy to use, quantitative results due to combination with strip reader	Non-determination of LOD and linear range	[61]
Colorimetric/LFIA	Immobilization of IL-6 antibody–AuNPs conjugates onto conjugated pad, biotinylated polyclonal IL-6 antibody and goat anti-mouse IgG antibody at the T and C lines, respectively, and silver amplification	5 pg mL^−1^	5–1000 pg mL^−1^	Serum	High sensitivity and specificity, wide linear range, short detection time (15 min)	Complexity of use due to the use of silver amplification technique	[33]
Colorimetric/solutions-based	Sandwich immunocomplex formation of Fe_3_O_4_-Ab_1_/IL-6/CeO_2_-Ab_2_ and oxidase activity of CeO_2_ nanospheres to catalyze the oxidation of OPD to yellow product oxOPD	0.04 pg mL^−1^	0.0001–10 pg mL^−1^	Serum	High sensitivity and specificity, wide linear range, satisfactory reproducibility, acceptable stability, and good accuracy	Several incubation steps and time-consuming procedure	[68]
Colorimetric/LFIA	Anti-IL-6 antibodies conjugated to AuNPs on conjugate pad, anti-IL6 and anti-mouse antibodies on T and C lines, respectively	0.38 ng mL^−1^	1.25–9000 ng mL^−1^	Plasma	Good sensitivity, high specificity, wide linear range, easy to use, good reproducibility, good shelf-life (6 months), short detection time (17 min)	-	[69]
Colorimetric/vertical flow assay	Spotting of anti-IL-6 capture antibody on nitrocellulose membrane, addition of IL-6, biotinylated anti-IL-6 detection antibody in AuNPs diluent and streptavidin-conjugated AuNPs	10 pg mL^−1^ in buffer 3.2 pg mL^−1^ in serum	10–10,000 pg mL^−1^	Serum	High sensitivity and specificity, wide linear range, high accuracy and reproducibility	Semi-quantitative results, requirement of several steps in addition of reagents	[70]
Colorimetric/LFIA	Application of selenium nanoparticles as labels for conjugation with anti-IL-6 detection antibody	0.1 ng mL^−1^	-	-	Good sensitivity, high specificity, good reproducibility	Non-determination of linear range, non-application in real samples	[71]

* nr: not reported.

**Table 4:** Representative examples of recently developed fluorescent-based optical biosensors for the detection of IL-6.

Type of Biosensor	Strategy of Detection	LOD	Linear Range	Matrix	Advantages	Disadvantages	Ref.
Fluorescence/LFIA	A double-antibody sandwich immunofluorescent assay based on EuNPs combined with lateral flow immunoassay	0.37 pg mL^−1^	2–500 pg mL^−1^	Serum	High sensitivity and specificity, wide linear range, high reliability, acceptable reproducibility, short detection time (15 min)	Need a reading device	[32]
Fluorescence/FRET	A FRET-based aptasensor using nitrogen-doped carbon quantum dots and gold nanoparticles	0.82 pg mL^−1^	1.5–5.9 pg mL^−1^	Serum	High sensitivity and specificity, simple procedure	Narrow linear range, long incubation time (1 h), Need spectrofluorimeter to read the results, no evaluation of reproducibility	[79]
Fluorescence/fiber optic	Coating of streptavidin-modified fiber surface with biotinylated capture antibodies and conjugation of detection antibodies with three different fluorescent magnetic beads	12.5 pg mL^−1^	12.5–200 pg mL^−1^	Serum	High sensitivity and specificity	Narrow linear range, long incubation time (2 h), need a microscope to obtain results and then perform image analysis, no evaluation of reproducibility	[83]
Fluorescence/SPFS	Sandwich immunoassay based on captured antibody immobilization on PDA layer and its complex formation with IL-6 and fluorescent labelled detection antibody	2 pg mL^−1^	2–2372 pg mL^−1^	Serum	High sensitivity and specificity, wide linear range	Requirement of several steps in addition of reagents, time consuming process, no evaluation of reproducibility	[84]
Fluorescence/LFIA	Quantum dots (QDs)-based LFIA strip by conjugating CdSe/ZnS QDs to the IL-6 antibody and spraying onto conjugate pad, immobilization of captured antibody IL-6 mAb2 and rabbit anti-mouse IgG onto T and C lines, respectively	1.995 pg mL^−1^	10–4000 pg mL^−1^	Serum	High sensitivity and specificity, wide linear range, simple analysis, high accuracy, high stability of strips at high temperature, high precision and acceptable reproducibility, short detection time (18 min)	Need a reading device	[85]
Fluorescence/LFIA	Application of CdSe QDs as label for conjugation with detection antibody and utilization of two image processing software for quantification of results	1.38 and 2.28 nM	0–20 nM	Serum	High sensitivity and selectivity, good reproducibility, multiplex assay	Narrow linear range, need a reading device	[86]
Fluorescence	Quenching of aptamer–fluorophore conjugate by adsorption to the graphene oxide outer layer of graphene oxide/nickel/platinum nanoparticle micromotor (MM) in the absence of IL-6 and fluorescence recovery by the aptamer- fluorophore separating from the MMs in the presence of IL-6	0.02 pg mL^−1^	0.07–1000 pg mL^−1^	Serum	High sensitivity and specificity, wide linear range, the need for small sample volume	No evaluation of reproducibility, long incubation time (30 min)	[87]

**Table 5:** Representative examples of recently developed SERS-based optical biosensors for the detection of IL-6.

Type of Biosensor	Strategy of Detection	LOD	Linear Range	Matrix	Advantages	Disadvantages	Ref.
SERS/label free	Aptamer-functionalized AuNPs array based on analyzing the change in the I_660_/I_736_ ratio of the SERS intensity derived from the guanine and adenine bases in the aptamer sequence	0.8 pM	10^−12^–10^−7^ pM	Mice serum	High sensitivity and specificity, wide linear range, label-free assay, good reproducibility	The need for Raman spectroscopy device, short-term stability of SERS substrate	[102]
SERS/label-based	A sandwich immunoassay based on complex formation between Au@Fe_3_O_4_ nanoring functionalized with antibodies, IL-6, and AgNPs functionalized with 4-MBA and antibodies	0.028 pg mL^−1^	0.1–1000 pg mL^−1^	Serum	High sensitivity and specificity, wide linear range, good stability of Ag@ 4-MBA@Antibody	Long incubation time (30 min), no evaluation of reproducibility	[103]
SERS/label-based	A self-calibrating aptasensor using immobilization of aptamer and its complimentary strand modified with Cy3 on the surface of Au@MBN@AgNPs	0.056 pg mL^−1^	10^−9^–10^−5^ mg mL^⁻1^	Serum	High sensitivity and specificity, wide linear range, excellent accuracy and reliability	No evaluation of reproducibility, long incubation time (90 min)	[34]
SERS/label-based	Combining multisite functionalized magnetic nanomaterials with interference-free SERS nanotags 4-MP and EB for multi-target detection of IL-6 and PCT	0.584 pg mL^−1^	1–5000 pg mL^−1^	Serum	High sensitivity and specificity, wide linear range, high stability and reproducibility of SERS probe	Multi-step preparation of sample	[104]
SERS/label-based	The core–shell nanoparticles embedded with a Raman reporter Au@4MBA@AgNPs were coupled to the tracer antibody, while the biotin was coupled to the captured antibody to form an antibody–antigen–antibody sandwich structure; capturing of sandwich structure by streptavidin magnetic beads and detection by Raman	1.6 pg mL^−1^	0–1000 pg mL^−1^	Serum	High sensitivity and specificity, wide linear range, short detection time and simple operation, short incubation time -5 min), good repeatability and precision	-	[105]
SERS/label-based	Magnetic sandwich immunoassay using core–shell Au@Ag embedded with a Raman tag and coupled with the tracer antibody as immunoprobe, biotin coupled with a second antibody and magnetic bead coated with streptavidin	0.54 pg mL^−1^	0–1000 pg mL^−1^	-	High sensitivity and specificity, wide linear range	No evaluation of real samples	[106]
SERS/label-based	A microfluidic system based on sandwich immunocomplex formation between immobilized antibodies against IL-6 captured on a Ag-Au bimetallic SERS-active surface, IL-6 and AuNPs conjugated with Raman tag and detection antibody	2.3 pg mL^−1^	0–30 pg mL^−1^	Plasma	High sensitivity, high enhancement factor, high reproducibility and stability of SERS substrate	Narrow linear range	[107]

## Data Availability

Not applicable.

## References

[1] Alexandre L., Bendali A., Pereiro I., Azimani M., Dumas S., Malaquin L., Mai T.D., Descroix S. (2022). Modular microfluidic system for on-chip extraction, preconcentration and detection of the cytokine biomarker IL-6 in biofluid. Sci. Rep..

[2] Moreno C., Mueller S., Szabo G. (2019). Non-invasive diagnosis and biomarkers in alcohol-related liver disease. J. Hepatol..

[3] Shi J., Huo R., Li N., Li H., Zhai T., Li H., Shen B., Ye J., Fu R., Di W. (2019). CYR61, a potential biomarker of tumor inflammatory response in epithelial ovarian cancer microenvironment of tumor progress. BMC Cancer.

[4] Seki E., Schwabe R.F. (2015). Hepatic inflammation and fibrosis: Functional links and key pathways. Hepatology.

[5] Gabay C. (2006). Interleukin-6 and chronic inflammation. Arthritis Res. Ther..

[6] Wang C., Xin D., Yue Q., Wan H., Li Q., Wang Y., Wu J. (2023). A novel electrochemical IL-6 sensor based on Au nanoparticles-modified platinum carbon electrode. Front. Bioeng. Biotechnol..

[7] Metcalfe R.D., Putoczki T.L., Griffin M.D. (2020). Structural understanding of interleukin 6 family cytokine signaling and targeted therapies: Focus on interleukin 11. Front. Immunol..

[8] Khan M.A., Mujahid M. (2020). Recent advances in electrochemical and optical biosensors designed for detection of Interleukin 6. Sensors.

[9] Tanaka T., Narazaki M., Kishimoto T. (2014). IL-6 in inflammation, immunity, and disease. Cold Spring Harb. Perspect. Biol..

[10] McCrae L.E., Ting W.-T., Howlader M.M. (2022). Advancing electrochemical biosensors for interleukin-6 detection. Biosens. Bioelectron. X.

[11] Wolf J., Rose-John S., Garbers C. (2014). Interleukin-6 and its receptors: A highly regulated and dynamic system. Cytokine.

[12] Garbers C., Hermanns H.M., Schaper F., Müller-Newen G., Grötzinger J., Rose-John S., Scheller J. (2012). Plasticity and cross-talk of interleukin 6-type cytokines. Cytokine Growth Factor Rev..

[13] Unver N., McAllister F. (2018). IL-6 family cytokines: Key inflammatory mediators as biomarkers and potential therapeutic targets. Cytokine Growth Factor Rev..

[14] Aydın E.B., Aydın M., Sezgintürk M.K. (2021). A novel electrochemical immunosensor based on acetylene black/epoxy-substituted-polypyrrole polymer composite for the highly sensitive and selective detection of interleukin 6. Talanta.

[15] Alfinito E., Beccaria M., Ciccarese M. (2020). Biosensing cytokine IL-6: A comparative analysis of natural and synthetic receptors. Biosensors.

[16] Aydın E.B. (2020). Highly sensitive impedimetric immunosensor for determination of interleukin 6 as a cancer biomarker by using conjugated polymer containing epoxy side groups modified disposable ITO electrode. Talanta.

[17] Eulenfeld R., Dittrich A., Khouri C., Müller P.J., Mütze B., Wolf A., Schaper F. (2012). Interleukin-6 signalling: More than Jaks and STATs. Eur. J. Cell Biol..

[18] Heinrich P.C., Behrmann I., Haan S., Hermanns H.M., Müller-Newen G., Schaper F. (2003). Principles of interleukin (IL)-6-type cytokine signalling and its regulation. Biochem. J..

[19] Jones S.A., Scheller J., Rose-John S. (2011). Therapeutic strategies for the clinical blockade of IL-6/gp130 signaling. J. Clin. Investig..

[20] Tanaka T., Narazaki M., Kishimoto T. (2012). Therapeutic targeting of the interleukin-6 receptor. Annu. Rev. Pharmacol. Toxicol..

[21] Scheller J., Garbers C., Rose-John S. (2014). Interleukin-6: From basic biology to selective blockade of pro-inflammatory activities. Semin. Immunol..

[22] Ridker P.M., Rane M. (2021). Interleukin-6 signaling and anti-interleukin-6 therapeutics in cardiovascular disease. Circ. Res..

[23] Hijazi Z., Aulin J., Andersson U., Alexander J.H., Gersh B., Granger C.B., Hanna M., Horowitz J., Hylek E.M., Lopes R.D. (2016). Biomarkers of inflammation and risk of cardiovascular events in anticoagulated patients with atrial fibrillation. Heart.

[24] Sahibzada H.A., Khurshid Z., Sannam Khan R., Naseem M., Mahmood Siddique K., Mali M., Zafar M.S. (2017). Salivary IL-8, IL-6 and TNF-α as potential diagnostic biomarkers for oral cancer. Diagnostics.

[25] Dalal V., Kumar R., Kumar S., Sharma A., Kumar L., Sharma J.B., Roy K.K., Singh N., Vanamail P. (2018). Biomarker potential of IL-6 and VEGF-A in ascitic fluid of epithelial ovarian cancer patients. Clin. Chim. Acta.

[26] Papiris S.A., Tomos I.P., Karakatsani A., Spathis A., Korbila I., Analitis A., Kolilekas L., Kagouridis K., Loukides S., Karakitsos P. (2018). High levels of IL-6 and IL-8 characterize early-on idiopathic pulmonary fibrosis acute exacerbations. Cytokine.

[27] Vainer N., Dehlendorff C., Johansen J.S. (2018). Systematic literature review of IL-6 as a biomarker or treatment target in patients with gastric, bile duct, pancreatic and colorectal cancer. Oncotarget.

[28] Dennen P., Altmann C., Kaufman J., Klein C.L., Andres-Hernando A., Ahuja N.H., Edelstein C.L., Cadnapaphornchai M.A., Keniston A., Faubel S. (2010). Urine interleukin-6 is an early biomarker of acute kidney injury in children undergoing cardiac surgery. Crit. Care.

[29] de Oliveira Gomes C.G., de Andrade M.V.M., Guedes L.R., Rocha H.C., Guimarães R.G., Carvalho F.A.C., Vilela E.G. (2020). Evaluation of the biomarkers HMGB1 and IL-6 as predictors of mortality in cirrhotic patients with acute kidney injury. Mediat. Inflamm..

[30] Graterol Torres F., Molina M., Soler-Majoral J., Romero-González G., Rodríguez Chitiva N., Troya-Saborido M., Socias Rullan G., Burgos E., Paúl Martínez J., Urrutia Jou M. (2022). Evolving concepts on inflammatory biomarkers and malnutrition in chronic kidney disease. Nutrients.

[31] Potere N., Batticciotto A., Vecchié A., Porreca E., Cappelli A., Abbate A., Dentali F., Bonaventura A. (2021). The role of IL-6 and IL-6 blockade in COVID-19. Expert Rev. Clin. Immunol..

[32] Huang D., Ying H., Jiang D., Liu F., Tian Y., Du C., Zhang L., Pu X. (2020). Rapid and sensitive detection of interleukin-6 in serum via time-resolved lateral flow immunoassay. Anal. Biochem..

[33] Rahbar M., Wu Y., Subramony J.A., Liu G. (2021). Sensitive colorimetric detection of interleukin-6 via lateral flow assay incorporated silver amplification method. Front. Bioeng. Biotechnol..

[34] Huang Q., Chen X., Fan M., Ruan S., Peng S., You R., Chen J., Lu Y. (2023). SERS-based self-calibrating aptamer sensor for selective detection of IL-6. Sens. Actuators B Chem..

[35] Hosseini S., Vázquez-Villegas P., Rito-Palomares M., Martinez-Chapa S.O., Hosseini S., Vázquez-Villegas P., Rito-Palomares M., Martinez-Chapa S.O. (2018). Advantages, disadvantages and modifications of conventional ELISA. Enzyme-Linked Immunosorbent Assay (ELISA) from A to Z.

[36] Dolin H.H., Papadimos T.J., Stepkowski S., Chen X., Pan Z.K. (2018). A novel combination of biomarkers to herald the onset of sepsis prior to the manifestation of symptoms. Shock.

[37] Monosik R., Stredanský M., Sturdik E. (2012). Biosensors-classification, characterization and new trends. Acta Chim. Slov..

[38] Singh A.K., Mittal S., Das M., Saharia A., Tiwari M. (2023). Optical biosensors: A decade in review. Alex. Eng. J..

[39] Damborský P., Švitel J., Katrlík J. (2016). Optical biosensors. Essays Biochem..

[40] Kaur B., Kumar S., Kaushik B.K. (2022). Recent advancements in optical biosensors for cancer detection. Biosens. Bioelectron..

[41] Herrera-Domínguez M., Morales-Luna G., Mahlknecht J., Cheng Q., Aguilar-Hernández I., Ornelas-Soto N. (2023). Optical Biosensors and Their Applications for the Detection of Water Pollutants. Biosensors.

[42] Estevez M.-C., Otte M.A., Sepulveda B., Lechuga L.M. (2014). Trends and challenges of refractometric nanoplasmonic biosensors: A review. Anal. Chim. Acta.

[43] Chen Y., Yu Y., Li X., Tan Z., Geng Y. (2015). Experimental comparison of fiber-optic surface plasmon resonance sensors with multi metal layers and single silver or gold layer. Plasmonics.

[44] Springer T.S., Ermini M.L., Spacková B., Jablonku J., Homola J. (2014). Enhancing sensitivity of surface plasmon resonance biosensors by functionalized gold nanoparticles: Size matters. Anal. Chem..

[45] Szymanska B., Lukaszewski Z., Oldak L., Zelazowska-Rutkowska B., Hermanowicz-Szamatowicz K., Gorodkiewicz E. (2022). Two Biosensors for the Determination of Interleukin-6 in Blood Plasma by Array SPRi. Biosensors.

[46] Mobed A., Shakouri S.K., Dolati S. (2020). Biosensors: A novel approach to and recent discovery in detection of cytokines. Cytokine.

[47] Chen P., Chung M.T., McHugh W., Nidetz R., Li Y., Fu J., Cornell T.T., Shanley T.P., Kurabayashi K. (2015). Multiplex serum cytokine immunoassay using nanoplasmonic biosensor microarrays. ACS Nano.

[48] He J., Zhou L., Huang G., Shen J., Chen W., Wang C., Kim A., Zhang Z., Cheng W., Dai S. (2022). Enhanced Label-Free Nanoplasmonic Cytokine Detection in SARS-CoV-2 Induced Inflammation Using Rationally Designed Peptide Aptamer. ACS Appl. Mater. Interfaces.

[49] Terada Y., Obara A., Briones J.C., Luo X., Espulgar W.V., Saito M., Takamatsu H., Tamiya E. (2023). Development of Nano–Micro Fused LSPR Chip for In Situ Single-Cell Secretion Analysis. Micromachines.

[50] Chou T.-H., Chuang C.-Y., Wu C.-M. (2010). Quantification of Interleukin-6 in cell culture medium using surface plasmon resonance biosensors. Cytokine.

[51] Zhu C., Luo X., Espulgar W.V., Koyama S., Kumanogoh A., Saito M., Takamatsu H., Tamiya E. (2020). Real-time monitoring and detection of single-cell level cytokine secretion using LSPR technology. Micromachines.

[52] Liu B., Zhuang J., Wei G. (2020). Recent advances in the design of colorimetric sensors for environmental monitoring. Environ. Sci. Nano.

[53] Geng Z., Miao Y., Zhang G., Liang X. (2022). Colorimetric biosensor based on smartphone: State-of-art. Sens. Actuators A Phys..

[54] Giorgi-Coll S., Marín M.J., Sule O., Hutchinson P.J., Carpenter K.L. (2020). Aptamer-modified gold nanoparticles for rapid aggregation-based detection of inflammation: An optical assay for interleukin-6. Mikrochim. Acta.

[55] Qian S., Cui Y., Cai Z., Li L. (2022). Applications of smartphone-based colorimetric biosensors. Biosens. Bioelectron. X.

[56] Alba-Patiño A., Russell S.M., Borges M., Pazos-Pérez N., Álvarez-Puebla R.A., de la Rica R. (2020). Nanoparticle-based mobile biosensors for the rapid detection of sepsis biomarkers in whole blood. Nanoscale Adv..

[57] Gumus E., Bingol H., Zor E. (2022). Lateral flow assays for detection of disease biomarkers. J. Pharm. Biomed. Anal..

[58] Sohrabi H., Majidi M.R., Fakhraei M., Jahanban-Esfahlan A., Hejazi M., Oroojalian F., Baradaran B., Tohidast M., de la Guardia M., Mokhtarzadeh A. (2022). Lateral flow assays (LFA) for detection of pathogenic bacteria: A small point-of-care platform for diagnosis of human infectious diseases. Talanta.

[59] Shirshahi V., Liu G. (2021). Enhancing the analytical performance of paper lateral flow assays: From chemistry to engineering. TrAC Trends Anal. Chem..

[60] Jøntvedt Jørgensen M., Holter J.C., Christensen E.E., Schjalm C., Tonby K., Pischke S.E., Jenum S., Skeie L.G., Nur S., Lind A. (2020). Increased interleukin-6 and macrophage chemoattractant protein-1 are associated with respiratory failure in COVID-19. Sci. Rep..

[61] Wang Y.-C., Lin S.-W., Wang I.-J., Yang C.-Y., Hong C., Sun J.-R., Feng P.-H., Lee M.-H., Shen C.-F., Lee Y.-T. (2022). Interleukin-6 test strip combined with a spectrum-based optical reader for early recognition of COVID-19 patients with risk of respiratory failure. Front. Bioeng. Biotechnol..

[62] Wang Z., Li Z., Zou Z. (2015). Application of binder-free TiO_x_N_1−x_ nanogrid film as a high-power supercapacitor electrode. J. Power Sources.

[63] Singh S. (2019). Nanomaterials exhibiting enzyme-like properties (nanozymes): Current advances and future perspectives. Front. Chem..

[64] Gao L., Zhuang J., Nie L., Zhang J., Zhang Y., Gu N., Wang T., Feng J., Yang D., Perrett S. (2007). Intrinsic peroxidase-like activity of ferromagnetic nanoparticles. Nat. Nanotechnol..

[65] Huang Y., Zhong H., Jiang C., Yang J., Zhang J., Zhao F., Liu C. (2024). Copper-based nanomaterials as peroxidase candidates for intelligent colorimetric detection and antibacterial applications. Particuology.

[66] Sun H., Zhou Y., Ren J., Qu X. (2018). Carbon nanozymes: Enzymatic properties, catalytic mechanism, and applications. Angew. Chem. Int. Ed..

[67] Nath I., Chakraborty J., Verpoort F. (2016). Metal organic frameworks mimicking natural enzymes: A structural and functional analogy. Chem. Soc. Rev..

[68] Peng J., Guan J., Yao H., Jin X. (2016). Magnetic colorimetric immunoassay for human interleukin-6 based on the oxidase activity of ceria spheres. Anal. Biochem..

[69] de Souza Sene I., Costa V., Brás D.C., de Oliveira Farias E.A., Nunes G.E., Bechtold I.H. (2020). A point of care lateral flow assay for rapid and colorimetric detection of interleukin 6 and perspectives in bedside diagnostics. J. Clin. Med. Res..

[70] Lei R., Arain H., Obaid M., Sabhnani N., Mohan C. (2022). Ultra-Sensitive and Semi-Quantitative Vertical Flow Assay for the Rapid Detection of Interleukin-6 in Inflammatory Diseases. Biosensors.

[71] Bradley Z., Coleman P.A., Courtney M.A., Fishlock S., McGrath J., Uniacke-Lowe T., Bhalla N., McLaughlin J.A., Hogan J., Hanrahan J.P. (2023). Effect of Selenium Nanoparticle Size on IL-6 Detection Sensitivity in a Lateral Flow Device. ACS Omega.

[72] Marshall J., Johnsen S. (2017). Fluorescence as a means of colour signal enhancement. Philos. Trans. R. Soc. B Biol. Sci..

[73] Kakkar S., Gupta P., Kumar N., Kant K. (2023). Progress in fluorescence biosensing and food safety towards point-of-detection (pod) system. Biosensors.

[74] Sharma A., Khan R., Catanante G., Sherazi T., Bhand S., Hayat A., Marty J. (2018). Designed strategies for fluorescence-based biosensors for the detection of mycotoxins. Toxins.

[75] Huang X., Aguilar Z.P., Xu H., Lai W., Xiong Y. (2016). Membrane-based lateral flow immunochromatographic strip with nanoparticles as reporters for detection: A review. Biosens. Bioelectron..

[76] Chang K., Zhu Q., Qi L., Guo M., Gao W., Gao Q. (2022). Synthesis and properties of nitrogen-doped carbon quantum dots using lactic acid as carbon source. Materials.

[77] Lin L., Luo Y., Tsai P., Wang J., Chen X. (2018). Metal ions doped carbon quantum dots: Synthesis, physicochemical properties, and their applications. TrAC Trends Anal. Chem..

[78] Sun Y., Shen C., Wang J., Lu Y. (2015). Facile synthesis of biocompatible N, S-doped carbon dots for cell imaging and ion detecting. RSC Adv..

[79] Mahani M., Faghihi-Fard M., Divsar F., Torkzadeh-Mahani M., Khakbaz F. (2022). Ultrasensitive FRET-based aptasensor for interleukin-6 as a biomarker for COVID-19 progression using nitrogen-doped carbon quantum dots and gold nanoparticles. Microchim. Acta.

[80] Buchegger P., Sauer U., Toth-Székély H., Preininger C. (2012). Miniaturized protein microarray with internal calibration as point-of-care device for diagnosis of neonatal sepsis. Sensors.

[81] Kapoor R., Wang C.-W. (2009). Highly specific detection of interleukin-6 (IL-6) protein using combination tapered fiber-optic biosensor dip-probe. Biosens. Bioelectron..

[82] Mowbray S., Amiri A. (2019). A brief overview of medical fiber optic biosensors and techniques in the modification for enhanced sensing ability. Diagnostics.

[83] Deng F., Qiao L., Li Y. (2022). A fluorescent immunosensor on optical fibre for the multiplex detection of proinflammatory cytokines. Sens. Bio-Sens. Res..

[84] Toma M., Tawa K. (2016). Polydopamine thin films as protein linker layer for sensitive detection of interleukin-6 by surface plasmon enhanced fluorescence spectroscopy. ACS Appl. Mater. Interfaces.

[85] Tang J., Wu L., Lin J., Zhang E., Luo Y. (2021). Development of quantum dot-based fluorescence lateral flow immunoassay strip for rapid and quantitative detection of serum interleukin-6. J. Clin. Lab. Anal..

[86] Ruppert C., Kaiser L., Jacob L.J., Laufer S., Kohl M., Deigner H.-P. (2020). Duplex Shiny app quantification of the sepsis biomarkers C-reactive protein and interleukin-6 in a fast quantum dot labeled lateral flow assay. J. Nanobiotechnology.

[87] Gordón J., Arruza L., Ibáñez M.D., Moreno-Guzmán M., López M.Á., Escarpa A. (2022). On the Move-Sensitive Fluorescent Aptassay on Board Catalytic Micromotors for the Determination of Interleukin-6 in Ultra-Low Serum Volumes for Neonatal Sepsis Diagnostics. ACS Sens..

[88] Azziz A., Safar W., Xiang Y., Edely M., de la Chapelle M.L. (2022). Sensing performances of commercial SERS substrates. J. Mol. Struct..

[89] Guillot N., de la Chapelle M.L. (2012). The electromagnetic effect in surface enhanced Raman scattering: Enhancement optimization using precisely controlled nanostructures. J. Quant. Spectrosc. Radiat. Transf..

[90] Gillibert R., Huang J.Q., Zhang Y., Fu W.L., de La Chapelle M.L. (2018). Explosive detection by surface enhanced Raman scattering. TrAC Trends Anal. Chem..

[91] Gillibert R., Triba M.N., de La Chapelle M.L. (2018). Surface enhanced Raman scattering sensor for highly sensitive and selective detection of ochratoxin A. Analyst.

[92] Guerrini L., Pazos-Perez N., Garcia-Rico E., Alvarez-Puebla R. (2017). Cancer characterization and diagnosis with SERS-encoded particles. Cancer Nanotechnol..

[93] Moisoiu V., Iancu S.D., Stefancu A., Moisoiu T., Pardini B., Dragomir M.P., Crisan N., Avram L., Crisan D., Andras I. (2021). SERS liquid biopsy: An emerging tool for medical diagnosis. Colloids Surf. B Biointerfaces.

[94] Jiang L., Hassan M.M., Ali S., Li H., Sheng R., Chen Q. (2021). Evolving trends in SERS-based techniques for food quality and safety: A review. Trends Food Sci. Technol..

[95] Mungroo N.A., Oliveira G., Neethirajan S. (2016). SERS based point-of-care detection of food-borne pathogens. Microchim. Acta.

[96] Tang H., Zhu C., Meng G., Wu N. (2018). Surface-enhanced Raman scattering sensors for food safety and environmental monitoring. J. Electrochem. Soc..

[97] Parambath J.B., Kim G., Han C., Mohamed A.A. (2023). SERS performance of cubic-shaped gold nanoparticles for environmental monitoring. Res. Chem. Intermed..

[98] Pérez-Jiménez A.I., Lyu D., Lu Z., Liu G., Ren B. (2020). Surface-enhanced Raman spectroscopy: Benefits, trade-offs and future developments. Chem. Sci..

[99] Safar W., Tatar A.-S., Leray A., Potara M., Liu Q., Edely M., Djaker N., Spadavecchia J., Fu W., Derouich S.G. (2021). New insight into the aptamer conformation and aptamer/protein interaction by surface-enhanced Raman scattering and multivariate statistical analysis. Nanoscale.

[100] Liu Y., Zhou H., Hu Z., Yu G., Yang D., Zhao J. (2017). Label and label-free based surface-enhanced Raman scattering for pathogen bacteria detection: A review. Biosens. Bioelectron..

[101] Gao S., Lin Y., Zhao X., Gao J., Xie S., Gong W., Yu Y., Lin J. (2022). Label-free surface enhanced Raman spectroscopy analysis of blood serum via coffee ring effect for accurate diagnosis of cancers. Spectrochim. Acta Part A Mol. Biomol. Spectrosc..

[102] Muhammad M., Shao C.-s., Huang Q. (2021). Aptamer-functionalized Au nanoparticles array as the effective SERS biosensor for label-free detection of interleukin-6 in serum. Sens. Actuators B Chem..

[103] Xie T., Xu D., Shang Y., Li Y., Gu Y., Yang G., Qu L. (2023). Highly sensitive SERS detection of IL-6 in serum by Au@ Fe3O4 nanoring-based sandwich immunoassay. Sens. Actuators B Chem..

[104] Wang Y., Guan M., Mi F., Geng P., Chen G. (2023). Combining multisite functionalized magnetic nanomaterials with interference-free SERS nanotags for multi-target sepsis biomarker detection. Anal. Chim. Acta.

[105] Wang X., Ma L., Sun S., Liu T., Zhou H., Liu X., Guan M. (2021). Rapid, highly sensitive and quantitative detection of interleukin 6 based on SERS magnetic immunoassay. Anal. Methods.

[106] Wang X., Ma L., Hu C., Liu T., Sun S., Liu X., Guan M. (2021). Simultaneous quantitative detection of IL-6 and PCT using SERS magnetic immunoassay with sandwich structure. Nanotechnology.

[107] Kamińska A., Winkler K., Kowalska A., Witkowska E., Szymborski T., Janeczek A., Waluk J. (2017). SERS-based immunoassay in a microfluidic system for the multiplexed recognition of interleukins from blood plasma: Towards picogram detection. Sci. Rep..

[108] Arano-Martinez J.A., Martínez-González C.L., Salazar M.I., Torres-Torres C. (2022). A framework for biosensors assisted by multiphoton effects and machine learning. Biosensors.

